# Inequalities in the frequency of free sugars intake among Syrian 1-year-old infants: a cross-sectional study

**DOI:** 10.1186/s12903-016-0287-8

**Published:** 2016-09-08

**Authors:** Easter Joury, May Khairallah, Wael Sabbah, Kanaan Elias, Raman Bedi

**Affiliations:** 1 Population and Patient Health, King’s College London Dental Institute, Denmark Hill Campus, Bessemer Road, London, SE5 9RS UK; 2 Oral Medicine Department, Faculty of Dentistry, Damascus University, Damascus, Syria; 3 Eastman Dental Institute, University College London, London, WC1X 8LD UK; 4 Centre for International Child Oral Health, King’s College London, 26-29 Drury Lane, Rooms 329-331, London, WC2B 5RL UK

**Keywords:** Social determinants of health, Socioeconomic factors, Health literacy, Health behaviour, Dietary sugars, Infant, Syria

## Abstract

**Background:**

High frequency of free sugars intake, during the first year of life is probably the greatest risk factor for early childhood caries. The latter is a global public health challenge. Very little is known about the social determinants of infant’s frequency of free sugars intake, particularly in low-income countries. Thus, the present study aimed to assess the association between the frequency of free sugars intake among 1-year-old Syrian infants and each of parents’ socioeconomic position (SEP), maternal frequency of free sugars intake and knowledge of infant’s oral health behaviour.

**Methods:**

Using a cross-sectional design, 323 1-year-old infants, attending vaccination clinics in 3 maternal and child health centres (MCHCs) in Damascus, Syria, were selected. A systematic random sampling was applied using the MCHCs’ monthly vaccination registries. The 3 MCHCs were located in affluent, moderate and deprived areas. Infants’ mothers completed a structured questionnaire on socio-demographics, infant’s and mother’s frequency of free sugars intake from cariogenic foods and beverages, and mother’s knowledge about infant’s oral health behaviour. Binary and multiple regression analyses were performed. The level of significance was set at 5 %.

**Results:**

The response rate was 100 %. Overall, 42.7 % of infants had high frequency of free sugars intake (>4times a day). Infants whose fathers were not working were more likely to have high frequency of free sugars intake. Similarly, infants whose mothers had low level of knowledge about infant’s oral health behaviour, or high frequency of free sugars intake were more likely to have high frequency of free sugars intake. The association between father’s occupation and infant’s frequency of free sugars intake attenuated after adjustment for mother’s knowledge and frequency of free sugars intake (adjusted OR = 1.5, 1.8, 3.2; 95%CI = 0.5–4.8, 1.1–3, 1.4–7.4; respectively).

**Conclusions:**

There are socioeconomic inequalities in the frequency of free sugars intake among Syrian 1-year-old infants. Integrated pre/post-natal interventions, targeting mothers from low SEP and aiming at reducing their free sugars intake and improving their knowledge about infant’s oral health behaviour, will potentially reduce socioeconomic inequalities in infant’s frequency of free sugars intake.

## Background

Establishing oral health promoting behaviour at an early stage of life, especially during the first year, should be the foundation for maintaining lifelong health behaviour [1]. Low frequency of free sugars intake is an essential aspect of oral health promoting behaviour [2]. Free sugars are defined as monosaccharides and disaccharides added to foods by the manufacturer, cook or consumer, and sugars naturally present in honey, syrups, fruit juices and fruit concentrates [3]. High frequency of free sugars intake is well-recognised as a determinant of early childhood caries (ECC) [4–6]. The latter is a global public health challenge [7], particularly in low-income countries. For example, Syria, like other countries in the Middle East, had a high prevalence of the disease (86 % in 2013) and a dmft value of 7.7 in preschool children aged 3–5 years.

Children’s frequency of free sugars intake is likely to be influenced by social factors such as parents’ socioeconomic position (SEP), mother’s knowledge of child’s oral health behaviour as well as mother’s own frequency of free sugars intake. Previous studies conducted on infants or pre-school children have demonstrated social inequalities in children’s sugar intake [8, 9]. Children from low SEP were more likely to have sugar-containing beverages than their counterparts from high SEP [8]. In a cross-sectional survey, mother’s perceived importance of controlling child’s sugary snacking was positively associated with child’s desirable sugary snaking behaviour [9].

Studies on infant’s sugar intake have focused on dietary patterns or feeding practices [10–12]. For example, mothers whose own diet was characterised by high intakes of sweets, chips, white bread, crisps were more likely to have infants who have high intakes of biscuits, chips, savoury snacks and bread. Conversely, mothers who complied with dietary recommendations, and who had high intakes of fruit, vegetables, wholemeal bread, rice and pasta were more likely to have infants who have comparable diets (high intake of fruit, vegetables and home-prepared foods) [10]. When the presence of cariogenic feeding practices was investigated, infants whose mothers had low education were at 70 % higher risk to have such cariogenic feeding practices than their counterparts whose mothers had high education [12]. Despite the importance of such findings in relation to infant’s dietary patterns, frequency of total free sugars intake holds paramount epidemiological and clinical relevance to dentistry [2, 3]. No previous study has investigated social inequalities in infant’s frequency of total free sugars intake.

Acknowledging the broader social determinants of frequent free sugars intake among Syrian infants and identifying the groups more likely to engage in this behaviour will potentially enable developing and initiating health promotion policies and interventions to establish desirable low frequency of free sugars intake, early in the life course. Furthermore, information on the social patterning of frequent free sugars intake among infants will help identifying target groups that need tailored health promotion interventions [13]. Therefore, the aims of the present study were: (1) to assess the association of the frequency of free sugars intake among Syrian 1-year-old infants with parents’ SEP, mother’s knowledge of infant’s oral health behaviour and her frequency of free sugars intake; and (2) to explore whether socioeconomic inequalities in infant’s frequency of free sugars intake could be explained by differences in mother’s knowledge of infant’s oral health behaviour and her frequency of free sugars intake.

## Methods

### Definition of high frequency of free sugars intake

High frequency of free sugar intake was defined as more than 4 occasions per day of cariogenic foods or beverages intake. The latter is considered the point at which caries would most likely develop [14].

### Study design, population and sampling

A cross-sectional study was conducted in 2012 on a sample of 1-year-old infants, attending vaccination clinics, at maternal and child health centres (MCHCs) in Damascus, Syria. Infants were selected from three MCHCs, by a systematic random sampling procedure using the MCHCs’ monthly vaccination registries (for first birthday vaccinations). The current study’s three MCHCs were located in three different areas: affluent, moderate and deprived areas, based on the average Real Estate value per square meter. The percentage of vaccination coverage in Damascus for infants aged 12–23 months was 89.4 % in 2011 [15]. This high percentage is explained by the fact that vaccination is mandatory in Syria and the child’s vaccination card is required for school registration.

### Sample size calculation

Determining the current study’s sample size required an estimate of the prevalence of infant’s high frequency of free sugars intake. There was no previous study conducted in Damascus city or Syria that investigated the frequency of free sugars intake in children under 3 years of age. Therefore, it was not feasible to determine *a priori* the aforementioned estimate. To address this, the present study conducted a pilot (on 60 infants; 20 from each MCHC) to draw an estimate of the prevalence of infant’s high frequency of free sugars intake. Based on the pilot the prevalence was 40 %. The latter was used to calculate the sample size.

A minimum sample size of 323 was estimated to demonstrate 2-fold or greater odds ratio in explanatory variables between infants with high frequency of free sugars intake and their counterparts with low frequency of free sugars intake. The calculation assumed no less than 40 % of high frequency of free sugars intake’s proportion. This calculation set the power of the test at 80 % and the level of significance at 5 %.

### Data collection procedures

Mothers of the selected infants were approached by the researcher (MK), in the waiting room, before baby’s vaccination. MCHCs usually run vaccination clinics once to twice a week in affluent/moderate and deprived areas, respectively. An informed letter explaining the study’s nature and general purpose was distributed to the mothers of selected infants. Thereafter, mothers were asked to complete a questionnaire in the waiting room before baby’s vaccination. Ten percent of the mothers were asked to complete the questionnaire again after baby’s vaccination to assess the reliability of collected data. This 10 % of mothers were also asked to complete an infant’s diet dairy to estimate the validity of infant’s frequency of free sugars intake question. A pamphlet on promoting child’s oral health was given to mothers after completing the questionnaire.

### Questionnaire

Mothers completed a piloted pre-tested structured questionnaire including information on socio-demographic background, infant’s frequency of free sugars intake and mother’s knowledge about infant’s oral health behaviour, as well as her frequency of free sugars intake.

### Infant’s and mother’s frequency of free sugars intake measure

Infant’s and mother’s frequency of free sugars intake was measured using two questions (one was related to the infant and the other one was related to the mother) as follows: how many times your baby/you consume sugary foods or drinks, such as, biscuits, chocolate, candies/confectionary, soft drinks [including squashes, juices and fizzy drinks], ice-cream, cakes, cookies, crisps, honey, sugary syrups or table sugar [added to meals, milk or teas, or used as a dip for baby’s pacifier], jam, halaweh [a tradition Syrian sweet food item], molasses, and other traditional sweets/desserts. The options were: (i) never, (ii) less than once a week. (iii) 1–6 times a week, (iv) 1–4 times a day, (v) more than 4 times a day. The cut off point, of 4 occasions or less per day of cariogenic foods or beverages, was used to dichotomise the frequency of free sugars intake into low [4 times or less a day] and high levels [more than 4 times a day].

### Parents’ socioeconomic position indicators

SEP information included parents’ occupation, working status and educational level. The reason for adopting a number of indicators was due to the fact that socioeconomic position (SEP) is not directly measurable but rather operationalised indirectly by a number of indicators [16]. Parents’ occupation included professional and manual occupations. Parents who do not work were grouped in one category. Education was measured by the highest qualification obtained [16] and dichotomised into two levels: high (post secondary school education) and low (secondary school or lower education).

### Mother’s knowledge about infant’s oral health behaviour measure

Mother’s knowledge about infant’s oral health behaviour was measured by 17 items developed by the current study, based on the British evidence-based guidelines for prevention of caries in children aged 0–3 years [2]. The items covered mother’s knowledge about: the importance of breastfeeding (1 item), appropriate time for bottle-feeding termination (around baby’s first birthday; 1 items), the best means to provide baby’s beverages (Sippy cup/ordinary cup; 1 item), baby’s ready food is not the healthiest choice for baby (1 item), the high content of added sugars in baby’s ready food (2 items), the potential harmful effect of adding sugars to baby’s food and beverages or offering confectionary snacks between meals (3 items), the best time to start cleaning baby’s teeth (since the first tooth erupted; 1 items), the best tool to clean baby’s teeth (baby’s toothbrush; 1 items), the best toothpaste to clean baby’s teeth (fluoride toothpaste; 1 items), the correct amount of toothpaste (a smear; 1 item); the frequency of cleaning baby’s teeth (at least twice a day; 1 item), the need for dentist consultation regarding professional preventive interventions (1 item), the importance of primary teeth (1 item), and the potential impact of dental caries on baby’s general wellbeing (1 item). The correct answers of the aforementioned knowledge items were summed into a final score. Final scores were dichotomised into high and low levels of mother’s knowledge about infant’s oral health promoting behaviour. Subjects who achieved a score that was equal to or higher than the median were assigned to the high level group whereas subjects who achieved a score that was below the median were assigned to the low level group.

### Data analysis

First, the presence of data entry errors was checked. Data were entered twice into separate files using the Statistical Package for Social Science software (SPSS version 20, IBM Corp., Armonk, NY, USA). When these two files were compared differences were highlighted. Any error identified was corrected by checking the infant's questionnaire.

Second, to assess the association between the current study explanatory variables (parents’ SEP and mother’s knowledge about infant’s oral health behaviour and her frequency of free sugars intake) and infant’s frequency of free sugars intake, binary logistic regression analyses were performed. Next, to assess the impact of mother’s knowledge about infant’s oral health behaviour and her frequency of free sugars intake on socioeconomic inequalities in infant’s frequency of free sugars intake, a set of logistic regression models adjusted for mother’s age, infant’s gender and family size, was performed [17]. The most significant SEP indicator was selected for model 1. Socioeconomic position is considered the most distal factor in the causal chain. In model 2, mother’s knowledge about infant’s oral health behaviour was added. In model 3, mother’s frequency of free sugars intake was added as the most proximal factor to infant’s frequency of free sugars intake. Finally, model 4 was adjusted for the centre’s level of deprivation to confirm the significance of identified social factors.

## Results

The response rate was 100 %. Test-retest reliability of collected data on socio-demographic characteristics, infants’ and mother’s frequency of free sugars intake and mother’s knowledge about infant’s oral health behaviour was good (rho = 1), indicating perfect agreement. Agreement was also excellent (rho = 1) between the questionnaire and infant’s diet diary regarding baby’s frequency of free sugars intake (high versus low frequency).

The 323 infants included 151 (46.7 %) males and 172 (53.3 %) females; 83.3 % of infants had a birth weight ≥2500 g.

Overall, 42.7 % infants had high frequency of free sugars intake (more than 4 times a day).

The results of binary logistic regression between the current study social explanatory variables and infant’s frequency of free sugars intake are summarised in Table 1.

**Table 1:** Binary logistic regression to predict infant’s high frequency of free sugars intake^a^

Variable	Base	Infant’s high frequency of free sugars intake (%)	OR (95 % CI)^d^
Father’s occupation			
Professional	224	96 (42.9)	1
Manual	74	26 (35.1)	0.7 (0.4–1.3)
Not working	19	13 (68.4)	2.9 (1.1–7.9)^b^
Missing	6		
Father’s education^e^			
High	91	40 (44)	1
Low	232	98 (42.2)	0.9 (0.6–1.5)
Missing	0		
Mother’s education^e^			
High	70	30(42.9)	1
Low	253	108 (42.7)	0.9 (0.6–1.7)
Missing	0		
Mother’s working status			
Working	56	29 (51.8)	1
Housewife	267	109 (40.8)	0.6 (0.4–1.2)
Missing	0		
Mother’s knowledge about infant’s oral health behaviour^f^			
High	139	11 (8.2 %)	1
Low	181	88 (48.6 %)	1.9 (1.2–2.9)^b^
Missing	3		
Mother’s frequency of free sugars intake^g^			
Low	287	112 (39 %)	1
High	36	26 (72.2 %)	4 (1.9–8.8)^c^
Missing	0		

^a^ *n* = 323

^b^ *P* < 0.050

^c^ *P* < 0.001

^d^OR (95 % CI): odds ratios (95 % confidence intervals)

^e^High level refers to post secondary school education, whereas low level refers to secondary school or lower education

^f^Measured by 17 items developed by the current study, based on the British evidence-based guidelines for prevention of caries in children aged 0–3 years [2]. High level refers to scores equal to or higher than the median, whereas low level refers to scores below the median

^g^High level refers to > 4 times a day, whereas low level refers to 4 times or less a day

Infants whose fathers were not working were almost three times more likely to have high frequency of free sugars intake than their counterparts whose fathers had professional occupation (Table 1). In contrast, infants whose mothers were housewives were less likely to have high frequency of free sugars intake than their counterparts whose mothers were working (Table 1). Infants whose mothers had low knowledge about infant’s oral health behaviour or high frequency of free sugars intake were two and four times more likely to have high frequency of free sugars intake than their counterparts whose mothers had high knowledge about infant’s oral health behaviour or low frequency of free sugars intake; respectively (Table 1).

Amongst SEP indicators, father’s occupation was the most significant SEP indicator at the 0.2 level. Thus, it was selected to enter the regression models.

Regression models, adjusted for mother’s age, infant’s gender and family size, are presented in Table 2. When mother’s knowledge about infant’s oral health behaviour and her frequency of free sugars intake were added in model 2 and 3, respectively, the association between father’s occupation and infant’s frequency of free sugars intake had progressively attenuated and lost its significance (Table 2). The association between infant’s frequency of free sugars intake, on the one hand, and mother’s knowledge about infant’s oral health behaviour and her frequency of free sugars intake, on the other hand, remained significant before and after adjustment.

**Table 2:** A set of logistic regression models ^c,d^ to predict infant’s high frequency of free sugars intake^a^

Variable	Adjusted OR (95 % CI)^e^			
	Model 1^c^	Model 2^c^	Model 3^c^	Model 4^d^
Father’s occupation				
Professional	1	1	1	1
Manual	0.7 (0.4–1.3)	0.7 (0.4–1.2)	0.7 (0.4–1.3)	0.7 (0.4–1.3)
Not working	3.1 (1.1–8.5)^b^	2.6 (0.9–7.3)	1.5 (0.5–4.8)	1.4 (0.4–4.4)
Mother’s knowledge about infant’s oral health behaviour^f^				
High		1	1	1
Low		1.9 (1.2–3.1)^b^	1.8 (1.1–3)^b^	1.8 (1.1–3)^b^
Mother’s frequency of free sugars intake^g^				
Low			1	1
High			3.2 (1.4–7.4)^b^	3.2 (1.4–7.4)^b^

^a^ *n* = 323

^b^ *P* < 0.050

^c^Model 1, 2 and 3 adjusted for mother’s age, infant’s gender and family size

^d^Model 4 additionally adjusted for centre’s level of deprivation

^e^OR (95 % CI): odds ratios (95 % confidence intervals)

^f^Measured by 17 items developed by the current study, based on the British evidence-based guidelines for prevention of caries in children aged 0–3 years [2]. High level refers to scores equal to or higher than the median, whereas low level refers to scores below the median

^g^High level refers to >4 times a day, whereas low level refers to 4 times or less a day

## Discussion

The current study demonstrated significant social inequalities in infant’s frequency of free sugars intake. Socioeconomic inequalities in infant’s frequency of free sugars intake were completey explained by mother’s frequency of free sugars intake and knowledge of infant’s oral health behaviour. To the best of our knowledge, this is the first study to investigate social inequalities in infant’s frequency of total free sugars intake and explore whether socioeconomic inequalities in infant’s frequency of free sugars intake would attenuate after adjusting for the effect of mother’s knowledge about infant’s oral health behaviour and her frequency of free sugars intake.

Inequalities in infant’s frequency of free sugars intake, identified in the current study, are in line with previous studies on infant’s dietary patterns and cariogenic feeding practices [10, 12]. It is worth mentioning that, amongst SEP indicators, mother’s education showed inconsistent associations with infant’s cariogenic feeding practices. For example, Feldens et al. reported that low maternal education was associated with higher risk of infant’s cariogenic feeding practices [12]. In contrast, Contreras et al. found that high maternal education was associated with higher infant’s consumption of highly processed snacks and sugar-sweetened beverages [11]. This variation in the relationship between maternal education and infant’s sugar consumption is probably attributed to the studied populations. Contreras et al. study was conducted on a rural population where mothers with high education might have more economic capacity to purchase cariogenic foods and beverages, without having appropriate knowledge about the risk of these foods and drinks on baby’s health [11]. Whereas Feldens et al. study was conducted on an urban population where mothers with high education might have better knowledge on the risk of cariogenic feeding practices and they might have more economic capacity to provide healthy alternatives to their babies [12].

In the current study, the association between father’s occupation and infant’s frequency of free sugars intake attenuated after adjustment for mother’s knowledge about infant’s oral health behaviour and her frequency of free sugars intake. This suggests a potential mediating role of mother’s knowledge and frequency of free sugars intake in explaining socioeconomic inequalities in infant’s frequency of free sugars intake.

The main limitation of the current study is its cross-sectional design. The significant associations found in this design do not imply causality. Furthermore, the direction of the association cannot be established. However, taking into account the context of the social determinants of health, it is unreasonable to assume that infant’s frequency of free sugars intake impacts on social factors such as father’s occupation. Despite efforts to minimise the possibility of recall bias by blinding the mothers to the current study’s specific scope, this possibility, as in any other retrospective study, could not be completely ruled out in the present study. The current study used one question to estimate the frequency of free sugars intake. Although such question is widely used in oral health surveys and despite the current study’s efforts to validate it against another dietary measure, more sophisticated instruments such as food frequency questionnaire might give a more precise estimate. Needless to say that such instruments are expensive to administer and time consuming. Another limitation pertains to the current study’s estimates’ precision. This is a common limitation of cross-sectional correlational studies that might not allow for a sufficient frequency distribution of different categories within a variable. In addition, correlational studies might not allow exploring all relevant associations. For example, the small numbers of working mothers did not allow exploring the impact of their type of occupation on their infant’s frequency of free sugars intake. Yet, the current study serves as a starting point for more expensive prospective longitudinal studies with a larger sample size to confirm the presence of social inequalities and determine the pathways through which these social inequalities are translated into differences in Syrian infant’s frequency of free sugars intake.

The current study’s findings can only be generalised to the current study’s Syrian infant’s population. The fact that vaccination is mandatory in Syria supports the generalisability of the current study’s findings to infants living in the catchment areas of selected MCHCs.

The importance of the present study’s findings is linked to their implications for child’s oral and general health in the short term and adult’s oral and general health in the long term. Inequalities in infant’s frequency of free sugars intake are likely to be translated into inequalities in early childhood caries and child’s obesity. For example, infant’s high frequency of sugary foods and beverages intake was independently associated with early childhood caries regardless of the frequency of sugars intake at later childhood age or oral hygiene practice [18, 19]. In addition, taste preference is established within the first 2 years of life [20]. Infants who develop a strong sweet taste preference such as that with free sugars might become more likely to have high frequency of free sugars intake in their later life stages [21, 22]. Therefore, it is very likely that such inequalities at an early stage of the life course might also lead to inequalities in adult’s frequency of free sugars intake and its related adulthood diseases such as dental caries, obesity and type 2 diabetes.

The policy, research and health promotion programmes implications of the present study’s findings could be summarised as follows: there is an urgent need for integrated oral health promotion interventions within the established Syrian national early childhood programmes, such as, breastfeeding and immunisation programmes. These whole population interventions should aim to reduce infant’s frequency of free sugars intake. They should also work on improving mother’s knowledge about infant’s oral health behaviour. To reduce social inequalities in infant’s frequency of free sugars intake, the aforementioned whole population programmes should be supported by other tailored oral health education and dietary interventions, targeting families from low SEP. Such interventions integrate the common risk factor approach into the social determinants framework [23], to promote infants’ general and oral health. Interventions at the pre-natal stage seem to be of paramount importance [24]. The World Health Organization (WHO) is called to review its recommendation on adding little quantities of sugary items to baby’s food [25].

## Conclusions

There are significant social inequalities in Syrian infant’s frequency of free sugars intake. Socioeconomic inequalities in infant’s frequency of free sugars intake might be explained by differences in mother’s knowledge about infant’s oral health behaviour and her frequency of free sugars intake. Thus, reducing these socioeconomic inequalities might be achieved by designing integrated oral health education and dietary interventions, within Syrian national early childhood programmes, targeting families from low SEP, to improve mother’s knowledge about infant’s oral health behaviour and reduce her frequency of free sugars intake.

## Abbreviations

SEP: Socioeconomic position
WHO: World Health Organization

## References

[1] Australian Dental Association. Caring for teeth for life. Australian Dental Association. 2007. http://www.pbcexpo.com.au/baby/baby-development/caring-for-baby-teeth/. Accessed 23 July 2014.

[2] Department of Health and British Association for the Study of Community Dentistry [BASCD] (2009). Delivering Better Oral Health: an evidence–based toolkit for prevention.

[3] World Health Organization and Food and Agriculture Organization (2002). Diet, nutrition and the prevention of chronic diseases; report of a joint WHO/FAO expert consultation.

[4] Harris R, Nicoll AD, Adair PM, Pine CM (2004). Risk factors for dental caries in young children: a systematic review of the literature. Community Dent Health.

[5] McDonagh MS, Whiting PF, Wilson PM, Sutton AJ, Chestnutt I, Cooper J (2000). Systematic review of water fluoridation. BMJ.

[6] Marinho VC (2009). Cochrane reviews of randomized trials of fluoride therapies for preventing dental caries. Eur Arch Paediatr Dent.

[7] Kassebaum NJ, Bernabé E, Dahiya M, Bhandari B, Murray CJ, Marcenes W (2015). Global burden of untreated caries: a systematic review and metaregression. J Dent Res.

[8] Wijtzes AI, Jansen W, Jansen PW, Jaddoe VW, Hofman A, Raat H (2013). Maternal educational level and preschool children’s consumption of high-calorie snacks and sugar-containing beverages: mediation by the family food environment. Prev Med.

[9] Adair PM, Pine CM, Burnside G, Nicoll AD, Gillett A, Anwar S (2004). Familial and cultural perceptions and beliefs of oral hygiene and dietary practices among ethnically and socio-economically diverse groups. Community Dent Health.

[10] Robinson S, Marriott L, Poole J, Crozier S, Borland S, Lawrence W (2007). Dietary patterns in infancy: the importance of maternal and family influences on feeding practice. Br J Nutr.

[11] Contreras M, Blandón EZ, Persson LÅ, Hjern A, Ekström EC (2015). Socio-economic resources, young child feeding practices, consumption of highly processed snacks and sugar-sweetened beverages: a population-based survey in rural northwestern Nicaragua. BMC Public Health.

[12] Feldens CA, Kramer PF, Sequeira MC, Rodrigues PH, Vitolo MR (2012). Maternal education is an independent determinant of cariogenic feeding practices in the first year of life. Eur Arch Paediatr Dent.

[13] Watt RG (2005). Strategies and approaches in oral diseaseprevention and health promotion. Bull World Health Org.

[14] Moynihan PJ, Kelly SA (2014). Effect on caries of restricting sugars intake: systematic review to inform WHO guidelines. J Dent Res.

[15] Central Bureau of Statistics. Family health survey in the Syrian Arab Republic, 2009-2010. 2011. http://www.cbssyr.sy/index-EN.htm. Accessed 2 Jan 2016.

[16] Liberatos P, Link BG, Kelsey JL (1988). The measurement of social class in epidemiology. Epidemiol Rev.

[17] Altman DG (1991). Practical statistics for medical research.

[18] Park S, Lin M, Onufrak S, Li R (2015). Association of sugar-sweetened beverage intake during infancy with dental caries in 6-year-olds. ClinNutr Res.

[19] Chaffee BW, Feldens CA, Rodrigues PH, Vítolo MR. Feeding practices in infancy associated with caries incidence in early childhood. Community Dent Oral Epidemiol. 2015;doi: 10.1111/cdoe.12158. 10.1111/cdoe.12158 PMC4491031 25753518

[20] Ventura AK, Worobey J (2013). Early influences on the development of food preferences. Curr Biol.

[21] Beauchamp GK, Moran M (1984). Acceptance of sweet and salty tastes in 2-year-old children. Appetite.

[22] Park S, Pan L, Sherry B, Li R (2014). The association of sugar-sweetened beverage intake during infancy with sugar-sweetened beverage intake at 6 years of age. Pediatrics.

[23] Watt RG, Sheiham A (2012). Integrating the common risk factor approach into asocial determinants framework. Community Dent Oral Epidemiol.

[24] Plutzer K, Spencer AJ (2008). Efficacy of an oral health promotion intervention in the prevention of early childhood caries. Community Dent Oral Epidemiol.

[25] World Health Organization. Consultation on infants and young children feeding; a participant guide. 2006.http://applications.emro.who.int/dsaf/dsa719.pdf?ua=1. Accessed 12 May 2015.

