# Erratum to: Inequalities in the frequency of free sugars intake among Syrian 1-year-old infants: a cross-sectional study

**DOI:** 10.1186/s12903-016-0305-x

**Published:** 2016-10-07

**Authors:** Easter Joury, May Khairallah, Wael Sabbah, Kanaan Elias, Raman Bedi

**Affiliations:** 1 Population and Patient Health, King’s College London Dental Institute, Denmark Hill Campus, Bessemer Road, London, SE5 9RS UK; 2 Oral Medicine Department, Faculty of Dentistry, Damascus University, Damascus, Syria; 3 Eastman Dental Institute, University College London, London, WC1X 8LD UK; 4 Centre for International Child Oral Health, King’s College London, 26-29 Drury Lane, Rooms 329-331, London, WC2B 5RL UK

## Erratum

Unfortunately, the original version of this article [1] was missing funding information within the funding section.

Please note that open access for this article was funded by King’s College London.
